# Modeling Immune Evasion and Vaccine Limitations by Targeted Nasopharyngeal *Bordetella pertussis* Inoculation in Mice

**DOI:** 10.3201/eid2708.203566

**Published:** 2021-08

**Authors:** Illiassou Hamidou Soumana, Bodo Linz, Kalyan K. Dewan, Demba Sarr, Monica C. Gestal, Laura K. Howard, Amanda D. Caulfield, Balázs Rada, Eric T. Harvill

**Affiliations:** University of Georgia, Athens, Georgia, USA

**Keywords:** *Bordetella pertussis*, upper respiratory tract infection, immune evasion, asymptomatic infection, bacteria, respiratory infections, vaccines

## Abstract

Conventional pertussis animal models deliver hundreds of thousands of *Bordetella pertussis* bacteria deep into the lungs, rapidly inducing severe pneumonic pathology and a robust immune response. However, human infections usually begin with colonization and growth in the upper respiratory tract. We inoculated only the nasopharynx of mice to explore the course of infection in a more natural exposure model. Nasopharyngeal colonization resulted in robust growth in the upper respiratory tract but elicited little immune response, enabling prolonged and persistent infection. Immunization with human acellular pertussis vaccine, which prevents severe lung infections in the conventional pneumonic infection model, had little effect on nasopharyngeal colonization. Our infection model revealed that *B. pertussis* can efficiently colonize the mouse nasopharynx, grow and spread within and between respiratory organs, evade robust host immunity, and persist for months. This experimental approach can measure aspects of the infection processes not observed in the conventional pneumonic infection model.

Less than a century ago, *Bordetella pertussis* was rampant worldwide, causing pertussis (whooping cough) that killed millions of persons every year, mostly infants and children (*1*). Whole-cell pertussis vaccines (wP), introduced in the mid-1950s, successfully controlled the disease, but concerns over side effects led many countries to replace wP vaccines with acellular pertussis (aP) vaccines in the mid-1990s (*2*). aP vaccines reduced side effects, but outbreaks of pertussis were still noted among highly aP-vaccinated populations (*3*), and the incidence of disease has been increasing among adults vaccinated with aP vaccines as children (*3*–*5*). In addition, experiments conducted with primates and rodents show that aP vaccines prevent the symptoms of disease but do not prevent the spread of the bacterium (*6*,*7*). There is now consensus among researchers that aP vaccines confer good but short-lived protective immunity against disease but much less protection against colonization, shedding, and transmission (*6*,*7*).

Most of our knowledge of *B. pertussis* has been learned from animal models of pneumonic infection that were developed during an era guided by Koch’s postulates (*8*–*19*). These animal experimental systems were designed to cause severe pathology and near-lethal virulence to simulate the most severe human disease. In pertussis models that emerged from this approach, large numbers of pathogen are introduced deep in the respiratory tract of animals, resembling extreme human infections in their severity and virulence but with more lung involvement than is generally clinically observed. In these models, high doses of *B. pertussis,* often 10^5^–10^6^ CFU, are delivered to the lungs of rodents (*20*,*21*). Larger primates, such as baboons, are inoculated by endotracheal intubation with even larger numbers, 10^8^–10^10^ CFU (*6*,*22*,*23*). 

High-dose pneumonic inoculations have provided several experimental benefits, including consistent colonization and growth of bacteria in the lungs, which induces severe pathology. Such inoculations served as assays to measure the contributions of individual virulence factors to severe disease and to develop effective vaccines. Delivery of large numbers of bacteria deep in the lungs predictably induces a vigorous and quantifiable immune response that begins to control infection within 2–3 weeks, reducing bacteria numbers below detectable levels within about 1 month (*6*,*24*) and providing an experimental system in which to develop and test vaccines to protect against such severe disease.

As valuable as conventional high-dose models have been, the bolus introduction of many bacteria deep into the lungs bypasses many key steps in the highly infectious catarrhal stage of pertussis, the prolonged period of early infection involving milder nonspecific upper respiratory tract symptoms. Of note, these aspects of early infection are most relevant to the current challenge of the ongoing circulation of *B. pertussis*. Indeed, recent work has revealed that a large proportion of human infections are asymptomatic and undiagnosed (*25*)*.* Assays that specifically measure how colonization, early growth, and immunomodulation contribute to shedding and transmission during the catarrhal stage of infection, before and perhaps independent of lower respiratory tract infection, are critical for development of vaccines that can prevent transmission.

We describe a novel nasopharyngeal infection model in mice that efficiently establishes *B. pertussis* infections that mimic human infections, beginning with low numbers of pathogens colonizing the upper respiratory tract. Nasopharyngeal infections in our model revealed crucial aspects of *B. pertussis*–host interactions not observed in conventional pneumonic infection models and successfully demonstrating the failure of aP vaccines to prevent nasopharyngeal colonization. This nasopharyngeal infection system allows mechanistic study of several aspects of the early infectious process that usually are obscured by conventional pneumonic challenge. In addition, the model provided assays that are likely to be useful for development of new and improved vaccines to prevent *B. pertussis* colonization and transmission.

## Materials and Methods

### Bacterial Cultures and Inocula Preparation

We grew *B. pertussis* strain 536, a derivative of strain Tohama I, as previously described (*12*). We then pelleted bacteria by centrifugation and resuspended it in phosphate-buffered saline (PBS) to an optical density of 600 nm (OD_600_) of 0.1 (≈10^8^ CFU/mL). We serially diluted bacteria in PBS to obtain 500 CFU in 5 μL PBS for low-dose–low-volume (LDLV) nasopharyngeal inoculation or 5 × 10^5^ CFU in 50 μL PBS for high-dose–high-volume (HDHV) pneumonic inoculation.

### Mouse Experiments

We housed C57BL/6 female mice from Jackson Laboratories (https://www.jax.org) in the specific pathogen-free facility at the University of Georgia (Athens, Georgia, USA). We diluted veterinary grade antimicrobial drugs, including enrofloxacin (Baytril; Bayer, https://www.bayer.com) and gentamicin (GentaFuse; Henry Schein, https://www.henryschein.com), for intranasal delivery in 10 µL PBS to mice anesthetized by using 10% isoflurane. We optimized the amount of antimicrobial drug delivered to a single dose of 45 μg enrofloxacin per mouse. Twelve hours after antimicrobial drug treatment, we delivered 500 CFU *B. pertussis *in 5 µL PBS for LDLV nasopharyngeal infections or 5 × 10^5^ CFU in 50 µL for HDHV pneumonic infections. Delivery of incula for both groups was by nasal inhalation under mild anesthesia. For vaccination experiments, we used intraperitoneal delivery, which is convenient and known to confer robust protection. In brief, we vaccinated 5-week-old mice on day 0 and gave a booster vaccine on day 28 by intraperitoneal injection of 200 µL PBS containing either wP vaccine (2 × 10^9^ CFU of *B. pertussis* Tohama I heat-killed at 65°C for 30 min) (*7*), or one tenth of a human dose of commercial aP (Adacel TdaP; Sanofi Pasteur, https://www.sanofi.us). We inoculated mice 2 weeks after the booster vaccination (day 42 post vaccination). At indicated time points, we euthanized mice by CO_2_ inhalation and excised nasal cavities, trachea, and lungs, which we homogenized in 1 mL sterile PBS by using Bead Mill 24 (Fisher Scientific, https://www.fishersci.com). We plated serial dilutions on Bordet-Gengou agar for bacterial enumeration.

### Flow Cytometry 

We prepared single-cell suspensions from collagenase-treated lungs, which we strained through 70 mm mesh and centrifuged through 44% Percoll (MP Biomedical, https://www.mpbio.com) in Gibco RPMI 1640 medium (Thermo Fischer Scientific, https://www.thermofisher.com), and then layered onto 67% Percoll in 1× PBS. We used TruStain FcX (Biolegend, https://www.biolegend.com) anti-mouse CD16/32 antibody to block Fc receptor cells and performed flow cytometry by using the LSR II system (BD Biosciences, https://www.bdbiosciences.com). We then stained surface markers with the antibodies used to sort neutrophils, T cells, B cells, and natural killer (NK) cells. We used the following Biolegend products from Thermo Fischer Scientific: for neutrophils, CD11b (CD11b Antibody, PE-Cyanine 7), CD115 (CD115 Antibody, APC), lymphocyte antigen complex 6 locus G (Lys6G Antibody, AF488); for T cells, CD45 (CD45 Antibody, Alexa Fluor-700), CD3 (CD3 Antibody-APC); for B cells, B220 (B220 Antibody-PE-Cy7), and NK cells, NK1.1 (NK1.1 Antibody-PE) (Appendix Figure 1). We analyzed data by using FACS Diva version 8.0.1 (BD Biosciences) and determined percentage viability by using Zombie Aqua (Biolegend) live-dead dye.

### Evaluation of Splenic Lymphocytes Responses

To analyze CD4 T cells and cytokines, including interleukin (IL) 17, IL-10, and IL-4, we collected spleens in ice chilled PBS (≈1°C–2°C) and then passed the mixture through a 40-µm cell strainer. We seeded 2 × 10^7^ cells in a 96-well plate and stained cells according to standard protocols (*26*). We acquired data in the LSR II (BD Bioscience) and analyzed data with FlowJo 10.0 by using a standard gating strategy (*27*). In brief, we used Ghost Dye Red 710 (Tonbo Biosciences, https://tonbobio.com) for determining live cells, then gated CD45+ for total leukocytes and Thy1.2+ for T cells. We used CD4+ cells to evaluate levels of intracellular IL-17, IL-4, and IL-10.

### *B. pertussis*–Specific Antibodies 

We quantified serum antibodies by ELISA using Corning Costar 96-well EIA microtiter plates (Thermo Fischer Scientific) coated with heat-killed *B. pertussis* grown to an OD_600_ of 0.600 in Stanier Scholte medium. We coated plates by using sodium-carbonate buffer (0.1 mmol/L at pH 9.5) overnight at 4°C (*28*). We considered the IgG titer to be the reciprocal of the lowest dilution in which we obtained an OD >0.1. 

We used 2-way analysis of variance and a paired 2-tailed Student *t*-test in Prism version 8.0.2 (GraphPad, https://www.graphpad.com) for statistical analyses between the pneumonic and nasopharyngeal groups. We performed animal experiments in accordance with recommendations in the Guide for the Care and Use of Laboratory Animals, National Research Council (https://www.nap.edu/read/12910). The study protocols were approved by the Institutional Animal Care and Use Committee at the University of Georgia (approval nos. A2016 02-010-Y3-A9 and A2016 04-019-Y3-A10).

## Results

### Nasopharyngeal Colonization 

**Figure 1:**
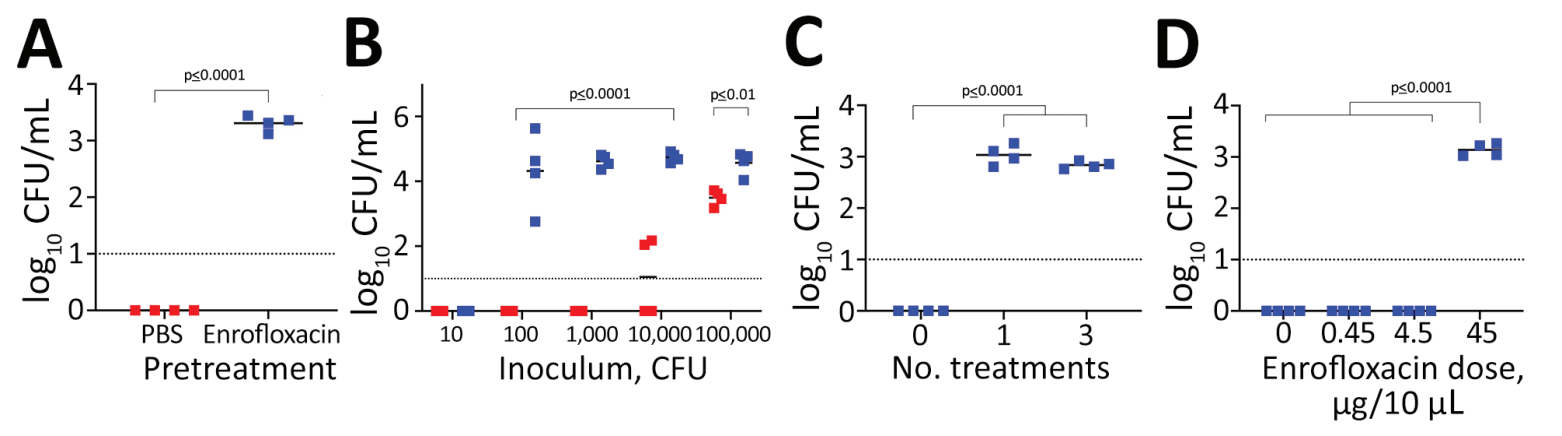
Susceptibility of mice to colonization by *Bordetella pertussis* after treatment with enrofloxacin. A, B) C57BL/6 mice were pretreated 3 times intranasally with 45 µg enrofloxacin in 10 µL (blue squares) or with phosphate-buffered saline (PBS; red squares) before being challenged with 5 µL PBS containing (A) 500 CFU of *B. pertussis*; or (B) *B. pertussis* ranging from 10–10,000 CFU. Colonization was assessed 3 days post inoculation by enumerating the number of *B. pertussis* CFU recovered from nasal cavities. C) Colonization and growth of *B. pertussis* at 500 CFU after 0, 1, and 2 pretreatments with 45 mg of enrofloxacin. D) *B. pertussis* colonization after intranasal enrofloxacin pretreatment at various doses. Each square represents a single biologic replicate. Dotted lines indicate limit of detection. Horizontal bars indicate mean.

*B. pertussis* generally is considered to be specialized to its human host and to have lost the ability to efficiently colonize other animals (*29*). However, in a previous investigation, we noted that resident nasal microbiota in mice create a barrier to colonization and that perturbing the microbiota with antimicrobial drugs permitted low numbers of *B. pertussis* to efficiently colonize the nasal cavities (*30*). To repeat this experiment and demonstrate improved ability to colonize mice, we intranasally treated groups of C57BL/6 mice (n = 4) 3 times, at 8-hour intervals, with either 45 μg enrofloxacin in 10 μL PBS or PBS only for the control group. Twelve hours after the last treatment, we intranasally delivered 500 CFU of *B. pertussis* in 5 µL of PBS to localize the inoculum within the nasal cavity. After 3 day, no *B. pertussis* were recovered from the nasal cavities of PBS-treated control mice, but we found all mice treated with antimicrobial drugs were colonized with thousands of CFUs of* B. pertussis*, indicating that enrofloxacin treatment facilitated *B. pertussis* colonization (Figure 1, panel A). We performed a similar experiment using gentamicin, which showed a similar increase in *B. pertussis* colonization, indicating that the effect is not limited to enrofloxacin (Appendix Figure 2). We also found no notable difference in respiratory tract colonization at days 3 and 7 between C57Bl6/J and BALBC/J mice that were treated with antimicrobial drugs and inoculated (Appendix Figure 3), indicating that nasopharyngeal colonization largely was independent of the genetic background between the 2 mouse strains.

Further optimization experiments (Figure 1, panels B, C, D) showed that pretreatment with antimicrobial drugs reduced the infective dose from 10,000 CFU in untreated mice to <100 CFU in treated mice (Figure 1, panel B). The threshold for successful nasal colonization was 4.5–45 µg of enrofloxacin. Even a single enrofloxacin pretreatment allowed *B. pertussis* to efficiently colonize mice (Figure 1, panels C, D). We settled on this relatively simple single enrofloxacin pretreatment and LDLV inoculation regimen as the experimental nasopharyngeal inoculation model.

### LDLV Nasopharyngeal Inoculation

**Figure 2:**
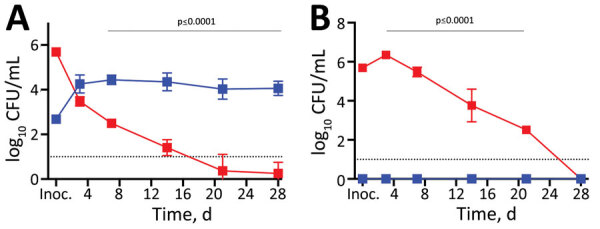
Growth and persistence of *Bordetella pertussis* in the nasal cavity of mice after low-dose–low-volume nasopharyngeal inoculation over time. C57BL/6 mice were inoculated intranasally with 500 CFU of *B. pertussis* in 5 µL phosphate-buffered saline for nasopharyngeal inoculations (blue squares) or 500,000 CFU in 50 µL for the pneumonic inoculations (red squares). The results were replicated in >4 study runs. *B. pertussis* colonization was assessed for the nasal cavities (A) and the lungs (B). Dotted lines indicate limit of detection. Error bars indicate SD of the mean. Inoc., inoculation.

We first assessed the course of infection in our model by comparing it with the conventional HDHV pneumonic model of *B. pertussis* infection. Groups of mice (4 per group) were either treated with enrofloxacin then nasopharyngeally inoculated with 500 CFU of *B. pertussis* in 5 µL PBS; or given the conventional HDHV pneumonic inoculation of 500,000 CFU of *B. pertussis* in 50 µL PBS. Both groups were sampled for >28 days (Figure 2, panel A). As usually observed in the HDHV pneumonic model, at day 3, *B. pertussis* had grown to large numbers in the lower respiratory tract of mice, but numbers were <10,000 CFU in the nasal cavities, and were undetectable in most HDHV mice by day 21, demonstrating more rapid clearance than is observed in human infections. In contrast, *B. pertussis* rarely reached the lungs of mice in the LDLV group (Figure 2, panel B), but *B. pertussis* numbers in the nasal cavity increased nearly 100-fold to ≈10,000 CFU and persisted at this level throughout the 28-day experiment (Figure 2, panel A). These data indicate that in the absence of lung infection, *B. pertussis* can efficiently colonize, grow, and persist in the nasopharynx.

### Host Immune Response

The colonization profile of the nasopharyngeal (LDLV) model revealed profound differences in the dynamics of the infection compared with the pneumonic (HDHV) model, suggesting very different interactions with host immunity. We and others previously have shown that the large bolus of *B. pertussis* delivered into the lungs in the pneumonic infection model rapidly activates both innate and adaptive immune components to generate a robust immune response that clears *B. pertussis* infection in ≈4 weeks (*11*,*12*). However, this infection model is unlike natural human infection because of the extraordinarily severe pneumonic disease, the robustness of the immune response, and the speed of bacterial clearance. In contrast, delivery of low doses of *B. pertussis* limited to the nasopharynx, more like natural exposure, resulted in localized growth in the upper respiratory tract, where the pathogen persisted at higher numbers for much longer. This finding led us to hypothesize that this more natural mode of infection might enable *B. pertussis* to grow more gradually, the way it would naturally, and thereby provide a model system to study how it might avoid unnecessary stimulation of host immunity to persist. To examine this hypothesis, we compared the relative proportions of major groups of immune cells in the lungs and nasopharyngeal washes on day 14 after HDHV pneumonic and LDLV nasopharyngeal inoculations.

**Figure 3:**
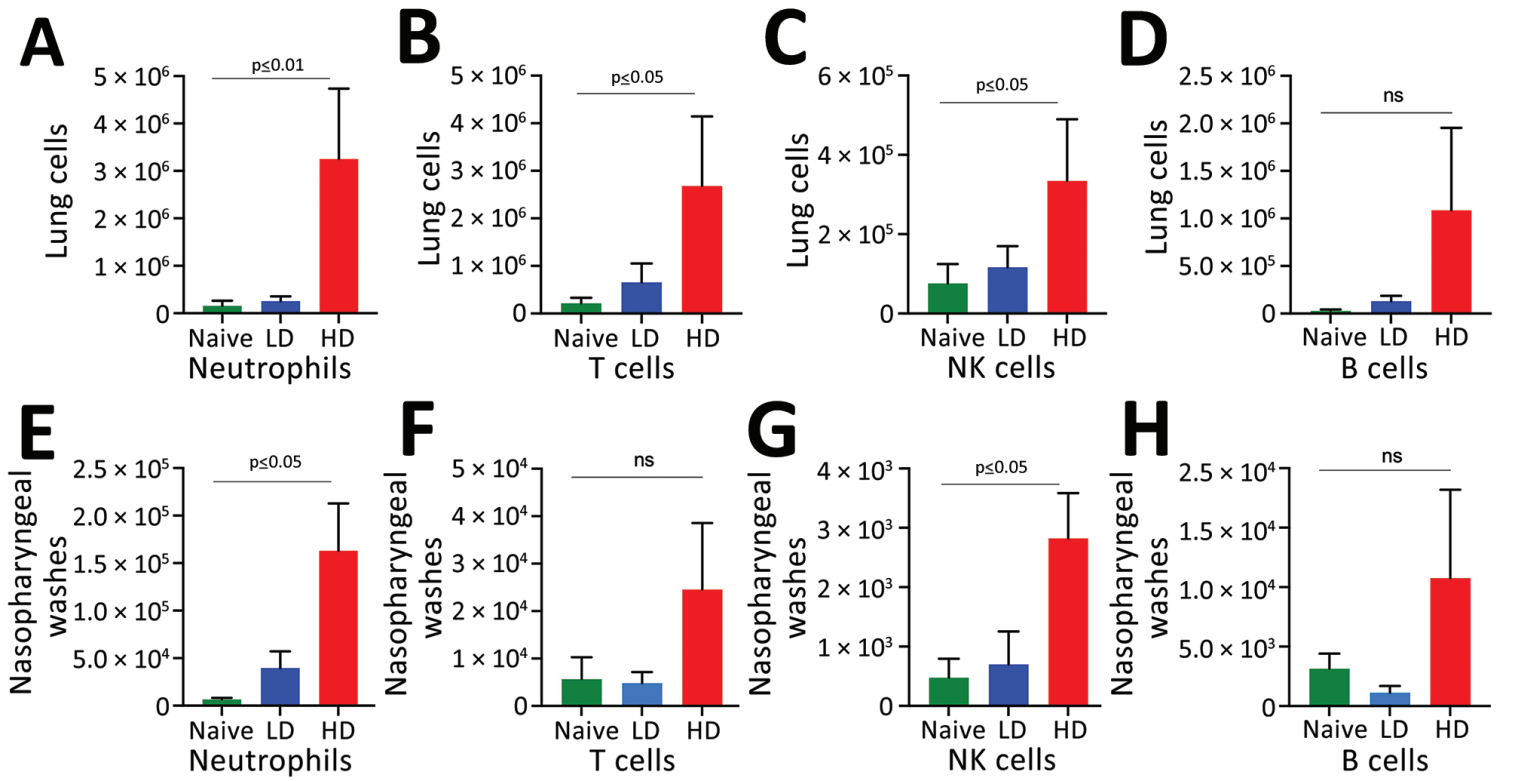
Host immune responses to LD and HD *Bordetella pertussis* inoculation. C57/Bl6 mice received LD of 500 CFU of *B. pertussis* in 5 µL phosphate-buffered saline (PBS) via nasopharyngeal inoculation or HD of 500,000 of *B. pertussis* CFU in 50 µL PBS via pneumonic inoculation. Naive control mice were inoculated with 50 µL of PBS. The study was conducted twice. A–D) Enumeration of immune cells in the lungs 14 days postinoculation. E–H) Enumeration of immune cells from nasopharyngeal washes. Error bars indicate SD for 4 biologic replicates HD, high-dose–high volume; LD, low-dose–low-volume; ns, no statistical significance.

**Figure 4:**
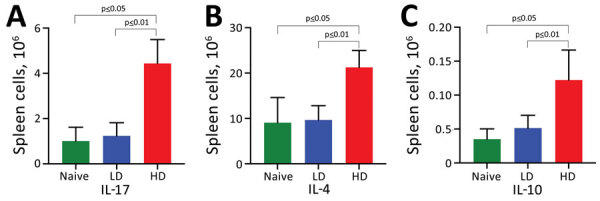
Host cytokine responses to LD nasopharyngeal inoculation and HD pneumonic inoculation of* Bordetella pertussis*. C57/Bl6 mice received LD of 500 CFU of *B. pertussis* in 5 µL phosphate-buffered saline (PBS) via nasopharyngeal inoculation or HD of 500,000 of *B. pertussis* CFU in 50 µL PBS via pneumonic inoculation. Naive control mice were inoculated with 50 µL of PBS. Splenocytes were isolated from mice at day 14 postinoculation. A) IL-17; B) IL-4; and C) IL-10. Error bars indicate SD for 4 biologic replicates; analysis was conducted once. HD, high-dose–high volume; IL, interleukin; LD, low-dose–low-volume.

Consistent with prior studies, the pneumonic infection model resulted in 5-fold to 50-fold increases in numbers of neutrophils (CD11b+/CD115–/Ly6G^high^), T cells (CD45+/CD3+), B cells (CD45+/B220+), and natural killer cells (CD45+/CD3–/NK1.1+) in the lungs (Figure 3, panels A–D) and in the nasal cavities (Figure 3, panels E–H) relative to control mice. By comparison, we detected only modest increases (<2-fold) among some immune cell populations in LDLV-inoculated mice, despite having even higher numbers of *B. pertussis* in the nose at the time. These observations indicate that *B. pertussis* can grow from small inocula to large numbers in the nasopharynx with minimal immune response. HDHV pneumonic inoculations also resulted in a robust systemic immune response indicated by the numbers of splenocytes with significant induction of IL-17, IL-4, and IL-10 compared with uninfected naive mice (Figure 4). But low-dose nasopharyngeal inoculation did not result in measurable increases in cytokines. Together these data reveal substantial differences in the immune response to pneumonic versus nasopharyngeal infection models.

### Persistent Nasopharyngeal Infection

**Figure 5:**
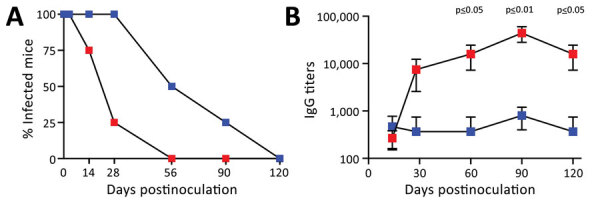
Comparison of serum IgG titers from mice receiving LD nasopharyngeal inoculation and HD pneumonic inoculation of* Bordetella pertussis*. Blue squares indicate LD mice; red squares indicate HD mice. C57/Bl6 mice received LD of 500 CFU of *B. pertussis* in 5 µL phosphate-buffered saline (PBS) via nasopharyngeal inoculation or HD of 500,000 of *B. pertussis* CFU in 50 µL PBS via pneumonic inoculation. Error bars indicate SD for 4 biologic replicates. A) Percentage of mice (4 per group) colonized on days 3, 7, 14, 28, 60, 90, and 120 following inoculations. Studies on days 3, 7, 14, and 28 were conducted 4 times; the 120-day experiment was conducted once. B) *B. pertussis* IgG titers in serum over time. HD, high-dose–high volume; LD, low-dose–low-volume.

A characteristic of pertussis in humans is the persistence of infection and disease lasting for many weeks or months; pertussis is also known as the 100-day cough. To compare persistence in the 2 contrasting infection models, we inoculated groups of C57Bl/6J mice to establish either nasopharyngeal (LDLV) or pneumonic (HDHV) infections. We then noted the presence or absence of *B. pertussis* in the nasal cavities (detection limit 10 CFU) on days 3, 7, 14, 28, 60, 90, and 120 postinoculation. For pneumonic infection models, the percentage of mice with bacteria recovered from the nasal cavities dropped from 100% on day 7 to 25% on day 28, after which bacteria were no longer detected (Figure 5, panel A). In contrast, LDLV nasopharyngeal inoculation resulted in more persistent infections; 100% of mice were still colonized at day 28 and 50% at day 60. Bacteria were still detected in 1/4 (25%) mice at day 90 and were only cleared from all mice 120 days after inoculation, highlighting the extraordinary persistence of this organism when delivered in more natural low dose and volume, and providing an experimental system in which to study its persistence.

As previously described for the HDHV pneumonic infection model, *B. pertussis* delivered to the lungs in large numbers induced a rapid increase in *B. pertussis* serum IgG titers to ≈10,000 by day 28 and to ≈20,000 by day 60 (Figure 5, panel B). As antibody titers rose, colonization levels dropped throughout the respiratory tract (Figure 5, panel A), consistent with the known roles of antibodies in clearing infection (*30*). Antibody titers continued to increase after the pathogen was cleared, contributing to the strong convalescent immunity associated with the conventional pneumonic model. In contrast, after LDLV nasopharyngeal inoculation, serum *B. pertussis* IgG levels were barely detectable even after months of persistent infection, reflecting the minimal induction, suppression, or both of host adaptive immunity by the pathogen. These lower antibody titers correlate with much slower control and clearance of infection in the nasopharyngeal infection model and in natural infections.

### Convalescent Immunity

Conventional HDHV pneumonic infections have been shown to induce robust protective immunity. However, LDLV nasopharyngeal inoculation resulted in more persistent infection and induced lower antibody titers, either because lower numbers of *B. pertussis* in the lungs are less immune stimulatory or because *B. pertussis* more effectively modulates the immune response when it follows this more natural course of infection. However, in both cases, infection eventually is cleared, indicating that adaptive immunity is generated and effective. To compare the relative efficacy of convalescent immunity induced by the 2 infection models, we examined the protection each conferred against subsequent challenge.

**Figure 6:**
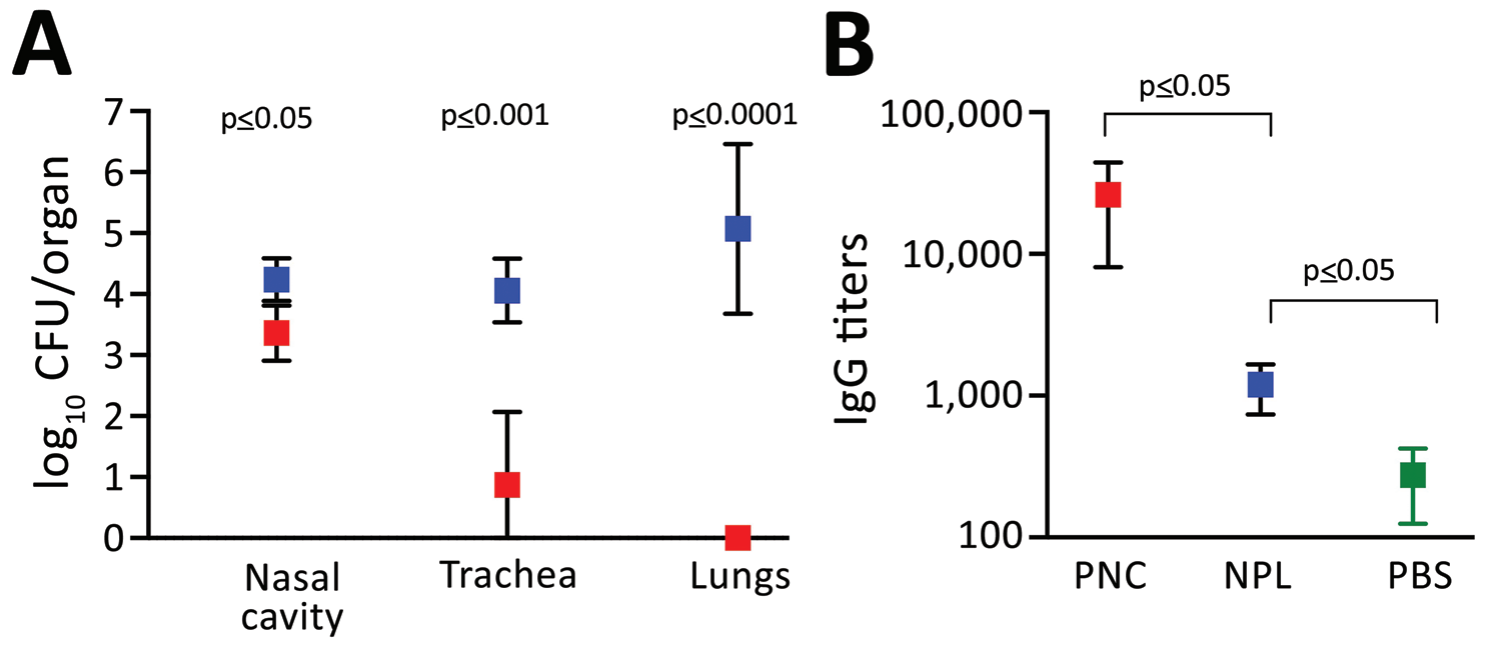
Risk for *Bordetella pertussis *reinfection after experimental nasopharyngeal infection of mice. C57Bl/6J mice were inoculated intranasally with 500 CFU of *B. pertussis* in 5 µL PBS for nasopharyngeal inoculations (blue squares) or 500,000 CFU in 50 µL for the pneumonic inoculations (red squares). The study was conducted twice. Values are the SD of 4 biologic replicates. A) Number of *B. pertussis* bacteria in respiratory organs on day 7 after pneumonic challenge. B) *B. pertussis* IgG titers (log scale) in the serum of mice challenged via PNC or NPL inoculation. Green represents naive mice inoculated with PBS. NPL, nasopharyngeal; PBS, phosphate-buffered saline; PNC, pneumonic.

Mice convalescing from prior pneumonic infection rapidly cleared a high-dose pneumonic challenge from the lungs and reduced numbers in the nasal cavity by >90% within 7 days (Figure 6, panel A), as previously documented (*32*,*33*). These mice showed no signs of disease, and bacterial numbers were far lower than those for unvaccinated mice challenged with the same dose (Figure 2, panel B), demonstrating that prior pneumonic infection confers protection against disease. In striking contrast, mice convalescing from prior low-dose nasopharyngeal inoculation had much higher numbers of *B. pertussis *in all respiratory organs. This finding shows that mice convalescing from nasopharyngeal infection fail to prevent subsequent colonization and bacterial growth when challenged with artificially large and deep lung pneumonic inoculation. These results agree with the corresponding serum antibody titers measured (Figure 6, panel B) and reveal profoundly different protective immunity induced by nasopharyngeal infection than described in previous studies that used the conventional HDHV pneumonic infection model (*24*,*32*).

### Vaccination Effects on Colonization

**Figure 7:**
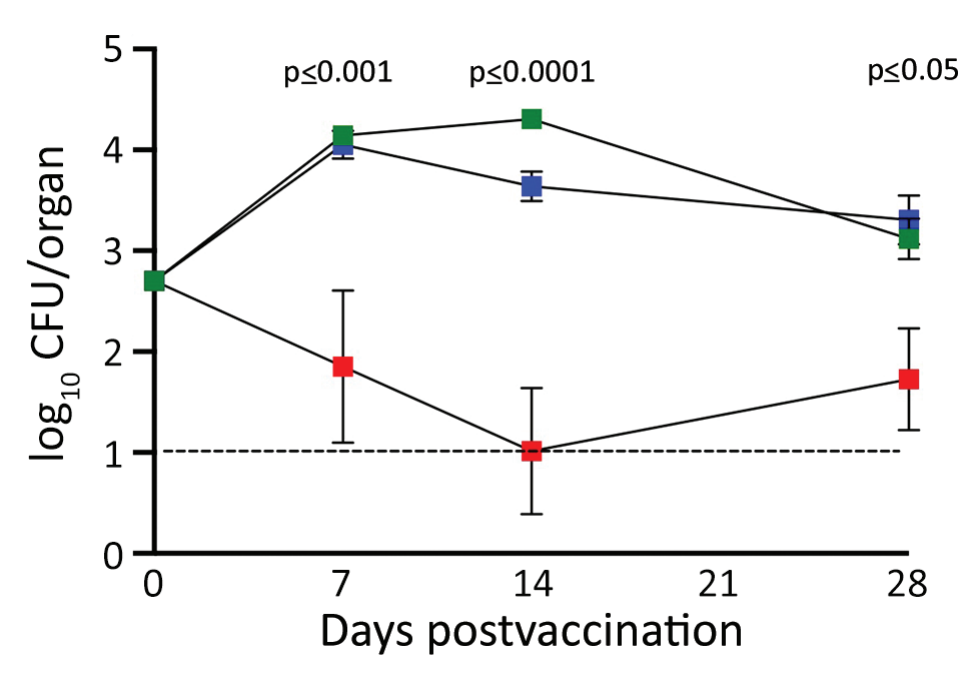
Comparison of nasal cavity colonization of *Bordetella pertussis *among experimentally infected mice after intraperitoneal vaccination with acellular pertussis (aP) or whole-cell pertussis (wP) vaccine. Graph compares colonization profiles over 28 days. Green squares indicate naive mice; blue squares indicate mice vaccinated with aP; red squares indicate mice vaccinated wP. Error bars indicate SD of the mean for 4 biologic replicates. The study was conducted twice; results are shown for a single experiment. Dotted line indicates limit of detection. p values indicate statistically significant differences between aP- and wP-vaccinated mice.

Although pneumonic models were central in developing aP vaccines that prevent severe disease, these assays of extreme pneumonic virulence failed to reveal the limited protection that aP vaccines provide against less severe upper respiratory tract colonization (*6*,*7*). Thus, these models did not predict the current problem of *B. pertussis* reemergence. Therefore, we set out to test whether the LDLV nasopharyngeal model might enable us to measure the failure of the aP vaccines and provide an assay system in which improved vaccines could be developed. For our vaccination experiments, we used the intraperitoneal delivery route, which is convenient and known to confer robust protection. Groups of mice that were vaccinated with either wP or aP vaccine, and unvaccinated control mice, were challenged via LDLV nasopharyngeal inoculation. wP-vaccinated mice were substantially protected against nasal colonization and had few or no bacteria remaining by day 7 after challenge (Figure 7). In contrast, *B. pertussis* colonized and grew in the nasal cavities of aP-vaccinated animals nearly as efficiently as in naive animals. These results demonstrate that aP vaccination fails to prevent nasopharyngeal colonization in this experimental system. This approach can measure the differences between wP and aP vaccines in this regard, providing an assay in which to evaluate various proposed new vaccines that might prevent colonization better than current aP vaccines (*34*,*35*).

## Discussion

Inoculating animals with high doses of *B. pertussis* delivered deep into the lungs (HDHV) induces severe pathology in the lower respiratory tract of rodents and baboons (*36*). Postmortem descriptions of lung pathology in 8 human infants who died from infantile pertussis revealed marked leukocytosis and pulmonary hypertension (*37*), features replicated in mouse and baboon pneumonic models (*36*,*38*,*39*), suggesting that these conventional pneumonic infection models reasonably replicate the most extreme form of human disease. However, these cases are extreme; pertussis generally is described as a disease of the upper respiratory tract that induces relatively little inflammation and histopathology (*40*,*41*) and often could occur with minimal symptoms and go undiagnosed (*25*). *B. pertussis* is highly infectious to humans, indicating that small numbers of bacteria landing in the upper respiratory tract can efficiently colonize, grow, and spread. However, conventional pneumonic infection models bypass the need to efficiently attach and establish the first microcolony, then grow and spread from there to other sites, potentially suppressing both the initial inflammatory response and the subsequent adaptive immune response. These aspects of the infectious process have not been well simulated in the HDHV pneumonic model, making it difficult to study and understand them.

We observed that localized application of antimicrobial drugs consistently enabled small numbers of *B. pertussis* to efficiently colonize, grow, and establish persistent infections in the nasopharynx of mice, mimicking the early stages of natural infection. Despite the efficient colonization and growth to higher and more sustained numbers in the nasal cavity, we detected only a modest (<2-fold) responses among immune cell populations. Furthermore, infections remained localized to the upper respiratory tract and rarely progressed to the lungs, agreeing with the notion that pertussis is primarily an upper respiratory tract infection. Of note, multiple contact tracing studies identify asymptomatic carriage as the likely source of human infections (*42*). In addition, the strong inflammatory responses and high antibody titers observed in pneumonic infection models are not routinely observed in most surveys of human infections (*43*–*45*).

Both wP and aP vaccines prevent severe pneumonic disease in HDHV pneumonic infection experimental models in rodents and primates, and both prevent severe disease in humans. However, consensus is growing that aP vaccines fail to prevent colonization and transmission, aspects of the infection process that are poorly simulated in the pneumonic infection model. Our findings for the novel LDLV nasopharyngeal infection system show that aP vaccines provide much less protection against colonization by small numbers of *B. pertussis* compared with wP vaccines. Thus, the LDLV nasopharyngeal infection model provides a complementary experimental system that enables the study of aspects of infection that are poorly mimicked in the HDHV pneumonic infection model. Further study of contemporary circulating *B. pertussis* strains in the context of low-dose nasopharyngeal infections could help define the factors that contribute to the diverse mechanisms by which *B. pertussis* evades immune responses. Such studies could elucidate how *B. pertussis* is able to colonize, grow, shed, and be efficiently spread from host to host within aP-vaccinated populations. Furthermore, our model can guide development of new vaccines that can overcome the limitations of current aP vaccines and better control the circulation of this reemerging pathogen.

Appendix Additional information on targeted nasopharyngeal *Bordetella pertussis* inoculation in mice.
