# Supplementary material for: Modeling Immune Evasion and Vaccine Limitations by Targeted Nasopharyngeal Bordetella pertussis Inoculation in Mice
Source: Emerg Infect Dis. 2021 Aug;27(8):2107–16. doi: 10.3201/eid2708.203566 (PMC8314809; doi:10.3201/eid2708.203566)
Supplement: Appendix — Additional information on targeted nasopharyngeal Bordetella pertussis inoculation in mice. [file 20-3566-Techapp-s1.pdf]

Article DOI: https://doi.org/10.3201/eid2708.203566

Modeling Immune Evasion and Vaccine Limitations by Targeted Nasopharyngeal *Bordetella pertussis* Inoculation in Mice

Appendix

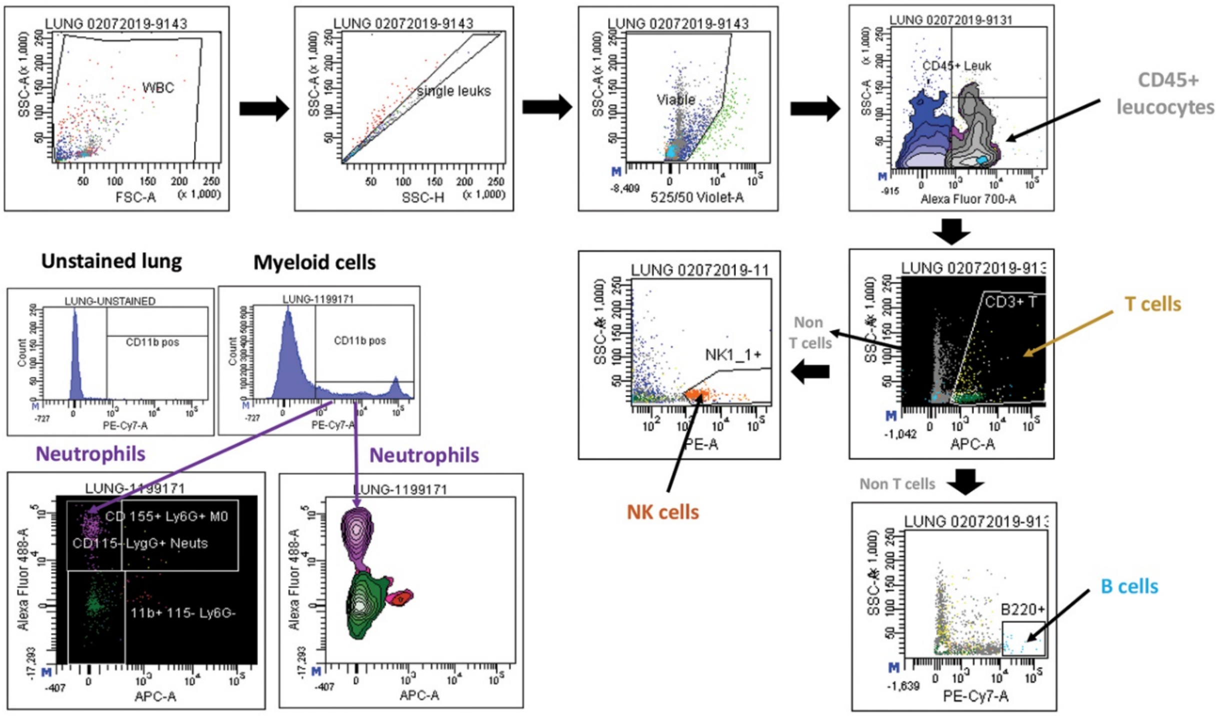

Appendix Figure 1. Gating strategy used to identify T cells, B cells, natural killer (NK) cells, and neutrophils in the lungs and nasal washes of *Bordetella pertussis*-infected mice. Leukocytes were gated based on forward scatter (FSC) and side scatter (SSC). Singlet cells were selected among the leukocytes; dead cells were removed based on Zombie-Aqua dye–positive cells. Total lymphoid cells (CD45+ cells) and total myeloid cells based on CD11b+ were gated among the viable cells of both the lungs and nasal washes. Neutrophils were gated among the CD11b+/CD115–/Ly6G^{high} cells. T cells were gated among the CD45+/CD3+ cells and B cells were gated among the CD45+/B220+ cells. NK cells were selected among CD45+/CD3–/NK1.1+, as indicated. Antibodies used for flow cytometry were Biolegend products (Thermo Fischer Scientific https://www.thermofisher.com). APC, allophycocyanin; APC-A, APC-Alexa Fluor Leuk, leuokocyte; PE, phycoerythrin; PE-A, PE-Alexa Fluor; pos, positive; +, positive.

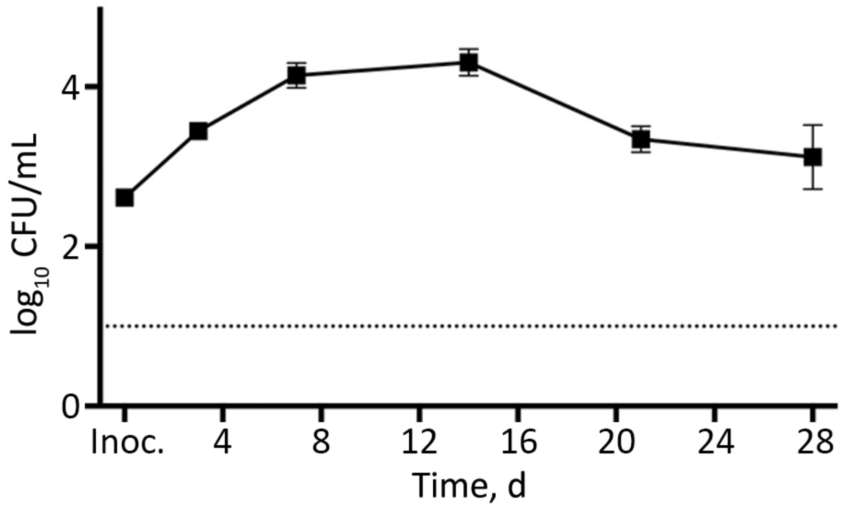

Appendix Figure 2. *Bordetella pertussis* colonization in nasal cavities of mice with after pretreatment with gentamicin. C57BL/6 mice (n = 4) were intranasally pretreated 3 times with 45 µg gentamicin in 10 µL of phosphate-buffered saline (PBS) before being inoculated with a low dose (500 CFU) of *B. pertussis* resuspended in a low volume (5 µL) of PBS. Colonization of nasal cavities was assessed by isolating the dorsal bones and internasal septum of the nasal cavities and homogenizing the tissue (3 days post inoculation). Bacterial CFUs were enumerated by growing serial dilutions of the homogenates on Bordet-Gengou agar supplemented with 10% sheep blood and 20 µg/mL streptomycin at 37°C. Dashed line indicates the limit of detection. Inoc., inoculation.

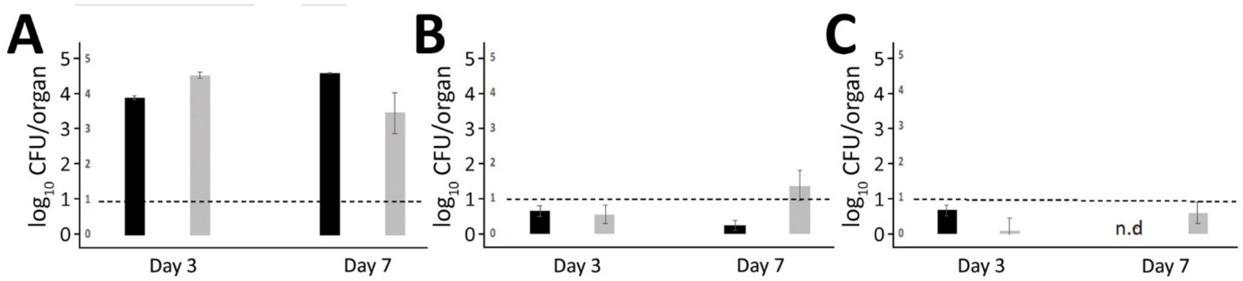

Appendix Figure 3. Colonization profile of *Bordetella pertussis* 536 in the respiratory tracts of C57BL/6J and BALB/CJ mice 7 days post inoculation. Graphs compare the number of CFUs of *B. pertussis* recovered on days 3 and 7 from the respiratory tracts (nasal cavities, trachea, and lungs) of C57BL/6J (black columns) and BALB/CJ (gray columns) mice intranasally inoculated with 500 CFU of *B. pertussis* in 5 µL of phosphate-buffered saline. No statistically significant differences were observed. Error bars indicate SD of the mean of 4 mice per group. Dashed lines represent the limit of detection. nd, no bacteria recovered from C57BL/6J mouse lungs on day 7.
